# Impact of Oral Nutritional Supplementation and Dietary Counseling on Outcomes of Linear Catch-Up Growth in Indian Children Aged 3–6.9 Years: Findings from a 6-Month Randomized Controlled Trial

**DOI:** 10.3390/children12091152

**Published:** 2025-08-29

**Authors:** Anuradha Khadilkar, Arati Ranade, Neelambari Bhosale, Swapnil Motekar, Nirali Mehta

**Affiliations:** 1 Jehangir Clinical Development Center Pvt. Ltd., Jehangir Hospital Premises, 32, Sassoon Road, Pune 411001, Maharashtra, India; arati.ranade@jcdc.co.in (A.R.); neelambari@jcdc.co.in (N.B.);; 2 PHARMA-STATS, 207, Symmers, B/w Sarkhej-Santipura Cross Road, Sanand-Sarkhej Rd, Sarkhej, Sarkhej-Okaf, Ahmedabad 382210, Gujarat, India

**Keywords:** undernutrition, stunting, picky eating, undernourished Indian children, oral nutritional supplement, dietary counseling, anthropometric measurement, protein-energy ratio

## Abstract

**Background/Objectives**: In India, according to the National Family Health Survey (NFHS-5), 36% of children under five years old are stunted, 19% are wasted, and 32% are underweight, indicating widespread undernutrition. **Methods**: A randomized controlled trial was conducted between August 2023 and May 2024 (CTRI/2023/04/051566), enrolling 223 undernourished Indian children, randomly assigned to the oral nutritional supplement (ONS) + dietary counseling (DC) (n = 111) arm or the dietary counseling (DC) arm (n = 112). This study recruited both male and female subjects with picky eating habits and with height-for-age (HAP) and weight-for-height percentiles (WHP) below the 25th percentile according to the WHO Growth Standards and Growth Reference. Outcomes assessed were anthropometric indices, dietary intake, sick days, and nutrient adequacy. Data were analyzed using ANCOVA, with statistical significance at *p* < 0.05. **Results**: At 6 months, the ONS + DC group showed significant improvements compared to DC in HAP (12.1 vs. −0.4, LS Mean difference [95% CI], 13.3 [11.13, 15.48], *p* < 0.0001), and WAP (9.7 vs. 2.3, LS Mean difference [95% CI] 7.9 [5.07, 10.78], *p* < 0.0001). MUACP significantly increased in the ONS + DC group (11.1 vs. −1.0 in DC, LS Mean difference [95% CI], 11.1 [5.28, 16.99], *p* < 0.0001). Dietary intake of carbohydrates, proteins, and energy was significantly higher in the ONS + DC group at 3 months, with sustained improvements at 6 months. By 6 months, the ONS + DC group showed a significantly higher protein-to-energy intake ratio compared to the DC group (0.0027 vs. −0.0003, LS Mean difference [95% CI] 0.00224 [0.00025, 0.00423], *p* = 0.0204). **Conclusions**: The addition of ONS + DC significantly improved linear catch-up growth outcomes in children at risk of undernutrition as a result of improved energy and nutrient intake and a higher protein-to-energy ratio.

## 1. Introduction

Malnutrition, encompassing both undernutrition and micronutrient deficiencies, is a critical global health concern, particularly among hospitalized children, where illness-related malnutrition often results from nutrient loss, increased energy expenditure, or impaired nutrient utilization, significantly impacting growth, development, and clinical outcomes [1,2]. In 2022, the global burden of undernutrition was significant; approximately 148.1 million children under the age of five were stunted, 45 million were wasted, and 13.6 million were severely wasted, with millions more experiencing micronutrient deficiencies [3].

In India, the situation is particularly concerning. As per the National Family Health Survey (NFHS-5), 36% of children under five were stunted, 19% wasted, and 32% underweight, reflecting both chronic and acute forms of undernutrition [4]. Factors driving malnutrition in India are multifaceted and include poverty, food insecurity, inadequate access to healthcare, and cultural dietary patterns. Traditional staple diets, often composed of cereals, rice, and limited protein sources, fail to provide a balanced nutritional profile, often lacking essential vitamins and minerals crucial for early childhood growth [5]. Economic constraints further limit families’ access to diverse, nutrient-dense foods, exacerbating these deficiencies [6,7]. As per the report on Nutrient Requirements-2020, these deficiencies are often referred to as “hidden hunger”, and they impact the health and development of children, who may consume enough calories but fail to receive balanced nutrition [8]. Undernutrition also weakens the immune system in children, making them more susceptible to infections and diseases, thus depleting the nutrient stores and impairing appetite, ultimately worsening the malnutrition [9].

Anthropometric measurements such as weight-for-age, height-for-age, and weight-for-height are key indicators used to assess children’s nutritional status. These are often expressed as Z-scores and percentiles [10]. Low scores reflect stunting, wasting, and underweight—markers of acute or chronic undernutrition. Additional indicators such as BMI-for-age and mid-upper arm circumference (MUAC)-for-age are also widely used [11]. Addressing nutritional deficiencies early can significantly improve these parameters and child health outcomes [12]. Various nutritional interventions have proven effective in addressing malnutrition and nutritional deficiencies among children under five [13].

Dietary counseling (DC) is also considered crucial for managing undernutrition, guiding the caregivers on nutritious food choices, balanced meals, and proper feeding schedules [14,15]. However, the effectiveness of DC alone in addressing undernutrition remains mixed and often depends on various factors, including the quality and intensity of the counseling provided, the caregiver’s motivation, and household constraints such as time restrictions that hinder full adherence to recommendations, resource availability, limited food diversity, financial constraints, and competing stresses [16,17]. The addition of nutritional supplementation along with DC will provide a more practical approach to addressing undernutrition and will also ensure that children receive essential nutrients even if dietary guidelines cannot be fully met [18].

Oral nutritional supplements (ONSs) have emerged as an effective intervention, providing concentrated nutrients that are often inadequate or lacking in children’s diets. ONS is designed to address the dietary gaps in young children and provide a balanced mix of macronutrients such as carbohydrates, proteins, and fats, along with vital micronutrients, including vitamins and minerals [19,20]. By delivering a balanced mix of macronutrients and micronutrients, these supplements support healthy weight gain and enhance overall growth metrics, including height-for-age, weight-for-age, and weight-for-height, which are the key indicators of nutritional status in undernourished children [21,22]. Protein in ONS aids tissue and muscle growth, while essential fats and carbohydrates provide energy for daily activity and healthy weight gain [23]. Immune-boosting nutrients such as vitamins A and C and zinc strengthen immune barriers and responses, thus helping children resist infections that can impede growth [24]. The use of ONS in combination with DC is supported not only through research but also seen in clinical practice models for managing pediatric malnutrition. Stepwise approaches, such as the clinical dietetic care pyramid, recommend ONS when food-based strategies alone are insufficient to meet nutritional requirements [25,26,27,28,29,30,31,32,33]. These principles provide the foundation for evaluating combined ONS and DC interventions in nutritionally at-risk children.

While these international studies support the long-term use of ONS + DC, evidence from India remains limited, particularly with respect to interventions extending beyond 3 months. Most Indian studies have either focused on short-term outcomes or assessed the effects of ONS and DC separately. There is, thus a pressing need for research evaluating the sustained impact of combined ONS + DC strategies in the Indian context. This study aims to compare the effect of ONS combined with DC versus DC alone over a 6-month period on key anthropometric outcomes, including weight-for-height and height-for-age percentiles, as well as nutritional intake in Indian children at nutritional risk.

## 2. Materials and Methods

### 2.1. Study Design and Study Population

This study was a 6-month, prospective, randomized controlled trial aimed at evaluating the effect of ONS + DC on growth in Indian children aged 3–6.9 years at risk of undernutrition. The study was designed to evaluate outcomes in comparison to a control group that received DC alone. Subjects were recruited from schools in the Pune region of Western Maharashtra. The study center was the Jehangir Clinical Development Centre Pvt. Ltd. in Pune, India.

Eligibility Criteria: This study is planned to recruit both male and female subjects aged 3.0 to 6.9 years. Subjects were eligible for inclusion if their height-for-age and weight-for-height (for ≤5 years) or BMI-for-age (for >5 years) were below the 25th percentile as per WHO Growth Standards [10]. Additional inclusion criteria included the ability to consume food and beverages orally, daily consumption of at least 200 mL of milk, and the presence of picky eating behaviors, defined as meeting at least two characteristics such as limited food variety, refusal to eat vegetables and/or foods from other food groups, unwillingness to try new foods, strong preferences, or disruptive mealtime behaviors [27,34]. Children also had to be in good general and mental health, which included a normal physical exam, stable vital signs, no acute illness or chronic conditions, and no behavioral or psychological concerns reported or observed at screening as determined by the investigator.

Exclusion Criteria: Subjects were excluded if they had known lactose intolerance, galactosemia, food allergies, developmental disorders, recent major illness or surgery, or any chronic or immunocompromising condition. Those on special diets, recent nutritional supplementation, or enrolled in another clinical or nutritional intervention within 30 days prior to screening were also excluded.

During the screening visit, participants and their parents/legal guardians were informed about the study, and all screening procedures were performed after written consent was obtained from the parents/legal guardians. The primary outcome was the change in height-for-age WHO percentile at 6 months compared to baseline. Secondary outcomes included changes in other anthropometric growth indicators at 3 and 6 months and the number of sick days and episodes, as well as changes in dietary intake and nutrient adequacy at 3 and 6 months relative to baseline. The exploratory outcomes of body composition and bone mineralization using Dual-Energy X-ray Absorptiometry (DXA), measured at baseline and 6 months only, will be presented in a separate publication. The study protocol was approved by the Institutional Ethics Committee of the Jehangir Clinical Development Centre Pvt. Ltd., and the trial was registered with the Clinical Trial Registry of India CTRI/2023/04/051566. The study was carried out in compliance with ICH E6(R2) “Guidance on Good Clinical Practice”, the Indian Good Clinical Practices Guideline, the ICMR Ethical Guidelines for Biomedical Research on Human Subjects, the ICMR National Ethical Guidelines for Biomedical and Health Research involving Human Participants (2017), and the Declaration of Helsinki.

### 2.2. Study Product

The study ONS (PediaSure; Abbott Healthcare Private Limited, Mumbai, India) provided a balanced profile of macronutrients and micronutrients essential for catch-up growth. At standard dilution, each 100 mL delivered 91 kcal of energy, 2.85 g of protein, 3.03 g of fat, and 13.10 g of carbohydrates, along with a wide range of vitamins and minerals. The formulation was also enriched with functional nutrients such as linoleic acid, alpha-linolenic acid, and prebiotic FOS, supporting energy metabolism and gut health, and nutrients that support growth such as arginine, vitamin K2, and casein phosphopeptides. Details of the nutritional composition are listed in Supplementary Table S1. The source of protein in the ONS is dairy-based, offering high-quality, easily digestible protein. Fat sources include soy oil, high oleic sunflower oil, and MCT oil, while carbohydrates are mainly derived from lactose, maltodextrin, and sucrose.

### 2.3. Study Procedure

As an open-label study, neither participants nor investigators nor the study team were blinded to treatment group allocation. Randomization was stratified by gender to recruit a target ratio of 50% female and 50% male, with at least 40% of either gender.

The study consisted of a screening and enrollment period (1 day) followed by a 6-month open-label study period. Children who met the eligibility criteria were randomized in a 1:1 ratio to either the intervention group (ONS + DC) or the control group (DC). Both groups received DC that emphasized balanced nutrition, while the intervention group also received two servings of the ONS daily throughout the study. The DC was provided by a qualified study nutritionist at baseline (Visit 1) and was reinforced during subsequent monthly follow-up visits throughout the 6-month study period. Each counseling session included a structured discussion tailored to the subjects’ age, nutritional status, physical growth parameters, and activity level. The focus was on educating caregivers about planning a balanced diet using locally available foods, appropriate portion sizes, and the importance of including all major food groups. To support the consistent implementation of DC, caregivers were provided with a subject diary to record daily food intake. Parents/LARs of subjects in the intervention arm were also trained to administer the study product (ONS), twice daily (45.5 g per serving) in addition to their regular diet, for which the subjects’ parent/LAR received dietary advice. The supplement was administered as a single serving of 225 mL, prepared by dissolving one 45.5 g sachet in 190 mL of water, stirred until completely dissolved, and consumed immediately. The actual dose consumed by the child was 225 mL, and each subject was instructed to take two servings per day. The nutritional composition of a 45.5 g serving of the study ONS is provided in Supplementary Table S1. One serving was administered at school by field staff, and the second was consumed at home, given by parents/LAR. The study intervention was consumed for a period of 180 days.

Participants attended a total of four scheduled visits during the study period: Visit 1 (Day 0, baseline), Visit 2 (1 month), Visit 3 (3 months), and Visit 4/End of Study (EOS, 6 months). The primary and secondary study endpoints were pre-specified to be assessed at 3 months (Visit 3) and 6 months (Visit 4/EOS). The 1-month visit (Visit 2) was included to monitor participant adherence, reinforce compliance with study procedures, and provide ongoing support, rather than for endpoint evaluation.

Baseline assessments at Visit 1 included Z-score and percentile estimation for anthropometric measurements (height, weight, BMI, weight-for-age, height-for-age, weight-for-height WHO, BMI-for-age, and mid-upper arm circumference) using WHO Anthro, version 3.2.2 and WHO Anthro plus software, version 1.0.4, bone mineral density (BMD) measured via DXA, dietary intake evaluation using a 24-h dietary recall in addition to recording medical history and concomitant medications, physical examinations and vital sign assessments. Subjects in both the groups received a diary to record dietary intake, sick days, and compliance with the study product (only for the interventional group). At Visit 2 (1 month from baseline) and Visit 3 (3 months from baseline), compliance with the dietary advice and study product was reviewed, DC was reinforced, and anthropometric parameters were reassessed alongside dietary intake using a 24-h recall. Compliance with the investigational product (IP) was assessed through diary reviews. The study coordinator will dispense a predefined quantity of the IP to the parent(s) or LAR of subjects in the interventional arm. At each visit, they will receive a sufficient amount of the IP to ensure continued use until the subsequent scheduled visit. Concomitant medications were reviewed and documented, and physical examinations and vital sign measurements were conducted.

At Visit 4 (6 months from baseline), which marked the end of the study visit, final reviews of diaries and anthropometric measurements were conducted, including a second DXA assessment. This structured visit schedule enabled comprehensive monitoring of dietary adherence, product compliance, growth, and overall health, facilitating an evaluation of the nutritional supplement’s impact on anthropometric parameters, dietary intake, and immunity. All anthropometric measurements were conducted by trained study personnel using standardized procedures as per WHO guidelines [35]. Weight was measured using calibrated digital scales to the nearest 0.1 kg, and height was measured using a stadiometer to the nearest 0.1 cm. MUAC was measured using non-stretchable measuring tapes. Growth velocity (in cm/month) was also calculated to assess linear growth over time.

The investigator ensured all adverse events (AEs), whether observed or reported, were accurately documented in medical records and AE forms.

### 2.4. Sample Size and Statistical Analysis

For sample size estimation, considering the efficacy variable of change in height-for-age percentile at 6 months, power = 80%, alpha = 0.05, a statistical two-sample *t*-test was used. A sample size of 90 in each group was estimated. With anticipated loss to follow-up of 30%, or 27 in each group, the final sample size to be considered for randomization and study totaled 234 in both groups. All analyses were conducted using R Version 4.3.1 or higher with a significance threshold set at a two-sided alpha level of 0.05. The anthropometric measurements were analyzed descriptively (i.e., number of non-missing observations, arithmetic mean, standard deviation, median, minimum, and maximum) and compared between the treatment groups using analysis of covariance (ANCOVA). The model included randomized treatment along with study center, gender, treatment*gender interaction, age (numeric), and baseline value as covariates. The estimated treatment effect was presented as the least square mean difference, with the corresponding two-sided 95% confidence interval (CI). The number of sick days is derived from an Adverse Event (AE) visit based on the date of visit and AE start date. Number of sick = AE End Date − AE start Date + 1. To evaluate nutrient adequacy in this study, the Estimated Average Requirement (EAR) cut-point method was considered, which is commonly recommended for assessing nutrient intake adequacy in population groups. In line with the method used in similar settings, this study employed a cut-off value of 80% of the RDA to determine nutrient adequacy [36]. The reference nutrient values used in this analysis were derived from the Indian Council of Medical Research–National Institute of Nutrition (ICMR–NIN) 2020 guidelines, which provide age- and sex-specific RDAs for macro- and micronutrients relevant to the Indian pediatric population [37]. Sensitivity analyses were additionally performed to determine the robustness of the results to outliers. Outliers were identified using the interquartile range method, based on changes in height (centimeters) from baseline at each visit, and excluded in sensitivity analyses for outcomes dependent on height measurements.

## 3. Results

**Figure 1:**
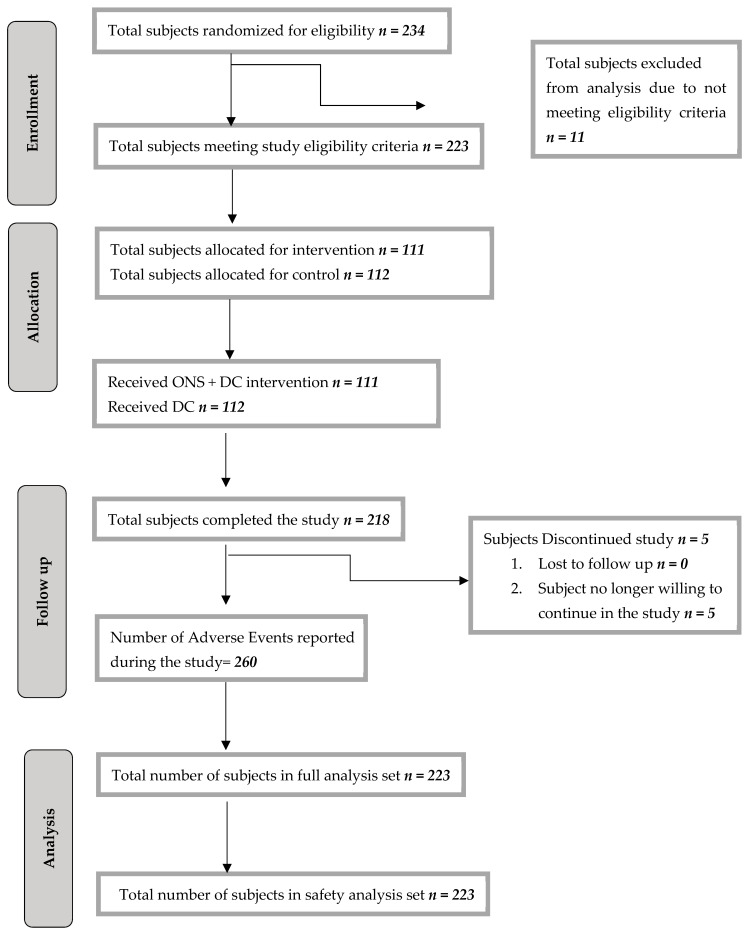
CONSORT flow diagram.

The study was conducted from 7 August 2023 to 10 May 2024, including the intervention period. Out of 234 subjects randomized, 223 met the eligibility criteria and were considered for final analysis. These were randomly assigned into two groups: 111 received ONS + DC, and 112 received only DC, as shown in the CONSORT flow diagram (Figure 1). Of the 223 enrolled, 218 completed the study (108 in the ONS group, 110 in the DC group). Five participants withdrew, citing unwillingness to continue. The results for height-related parameters were included following the completion of sensitivity analyses, ensuring the robustness and reliability of the observed outcomes.

Among the 223 subjects analyzed for safety, 112 (50.2%) were male and 111 (49.8%) were female. The participants, aged 3 to 6.9 years, had a mean age of 4.9 years. The overall population exhibited a mean height of 101.4 cm (5.7), body weight of 14.0 kg (1.3), and BMI of 13.5 kg/m^2^ (0.5), with height-for-age and weight-for-height percentiles of 8.1 (6.7) and 12.8 (6.5), respectively. Group-wise detail summary of demographics provided in Table 1.

### 3.1. Change in Mean Height and Weight

Table 2 shows that at baseline, the average height and weight were comparable between the ONS + DC group and the DC group. For height, the baseline mean was 101.0 (5.8) cm in the ONS + DC group and 101.7 (5.7) cm in the DC group. Over the study period, the ONS + DC group demonstrated significantly greater height gains compared to the DC group. By 6 months, the total height gain was 5.8 (1.5) cm in the ONS + DC group compared to 2.4 (1.0) cm in the DC group (*p* < 0.001).

For body weight, the baseline mean was 14.0 (1.3) kg in both the ONS + DC group and the DC group. By 6 months, the ONS + DC group achieved a total weight gain of 2.1 (0.9) kg compared to 0.9 (0.8) kg in the DC group (*p* < 0.001). These findings show that ONS + DC led to significantly greater improvements in height and weight compared to DC alone.

### 3.2. Height Indices and Proportionate Weight Indices

The changes in growth parameters over 3 and 6 months are presented in Table 3. At baseline, all anthropometric measures were comparable between the ONS + DC group and the DC group.

At 3 and 6 months, the ONS + DC group demonstrated significantly greater improvements in HAP, WAP, and MUACP anthropometric outcomes compared to the DC group alone.

For HAP, the mean change from baseline at 3 months was 10.0 (10.8) in the ONS + DC group, compared to 1.3 (4.8) in the DC group. The least-squares mean (LS Mean) difference between groups was 9.50 [95% CI: 7.49, 11.52], which was statistically significant (*p* < 0.0001). At 6 months, the mean change from baseline in the ONS + DC group further improved to 12.1 (11.3), while the DC group showed a decline of –0.4 (4.8). The between-group difference increased to 13.30 [95% CI: 11.13,15.48], again statistically significant (*p* < 0.0001).

In terms of WAP, the ONS + DC group showed a mean change from baseline of 9.0 (10.5) at 3 months, compared to 4.2 (7.1) in the DC group (LS Mean difference: 5.3 [95% CI: 2.5, 8.2]; *p* < 0.0001). At 6 months, the improvement in the intervention group was 13.7 (14.9) versus 2.3 (6.3) in the control group, with a significant between-group difference of 7.9 [95% CI: 5.0, 10.7]; *p* < 0.0001).

For WHP and BMI-for-age percentile, although improvements were seen in both groups, the between group differences at 3 and 6 months were not statistically significant.

For MUACP, improvements were observed in the ONS + DC group at both time points. At 3 months, the mean change from baseline was 9.8 (13.6) compared to 3.0 (7.6) in the DC group (LS Mean difference: 6.5 [95% CI: 0.8, 12.1]; *p* = 0.0113). At 6 months, the ONS + DC group improved by 11.1 (13.0), while the DC group declined by –1.0 (8.4), yielding a significant (LM mean difference of 11.1 [95% CI: 5.28, 16.9]; *p* < 0.0001).

Results of analyses using standardized Z-scores were similar to percentiles and are listed in Table 3.

In sensitivity analyses, excluding outliers based on changes in height from baseline, nine participants from the ONS + DC cohort were excluded. Results from sensitivity analyses remained unchanged, indicating that conclusions remain robust even after outlier exclusion (Supplementary Table S2).

### 3.3. Number of Sick Days and Sick Episodes at 3 and 6 Months

As presented in Table 4, there was no significant difference in the number of sick days between the ONS + DC and DC groups over the 6-month study period. The mean number of sick days showed a slight decrease in both groups (ONS + DC: 3.8 (1.7) to 3.6 (1.1) days; DC: 3.7 (1.5) to 3.2 (1.3) days), with the median remaining stable at 3.0 days across both time points in each group.

The percentage of children experiencing sick episodes in the ONS + DC group reduced from 30.6% at 3 months to 19.8% at 6 months. However, the percentage of children with sick episodes in only the DC group increased from 21.4% to 27.7% over the same period. Nevertheless, there were no significant differences in the rate ratios of sick episodes between groups at either time point.

### 3.4. Nutritional Intake at 3 and 6 Months

At baseline, nutritional intake was not different between groups. Table 5 shows that the ONS + DC group experienced significant improvements in carbohydrate, protein, and energy intake compared to the DC group.

At both 3 and 6 months, the ONS + DC group showed significantly greater improvements in carbohydrate and protein intake compared to the DC group. Specifically, the mean change from baseline (CFB) in carbohydrate intake at 3 months was significantly higher in the ONS + DC group, with an adjusted mean difference of 28.5 g/day (95% CI: 19.36, 37.7; *p* < 0.0001). This difference decreased but remained significant at 6 months (12.4 g/day; 95% CI: 3.2, 21.6; *p* = 0.0031) between groups.

Similarly, protein intake increased significantly in the ONS + DC group at both 3 months (mean difference: 6.4 g/day; 95% CI: 3.6, 9.3; *p* < 0.0001) and 6 months (mean difference: 4.1 g/day; 95% CI: 1.2, 6.9; *p* = 0.0014) compared to the DC group.

Energy intake also significantly improved at 3 months in the ONS + DC group (mean difference: 155.9 kcal/day; 95% CI: 96.3, 215.4; *p* < 0.0001), though the difference was not statistically significant at 6 months.

A small but statistically significant improvement in the protein-to-energy ratio was observed at 6 months in the ONS + DC group (mean difference: 0.0022; 95% CI: 0.0003, 0.0042; *p* = 0.0204). No statistically significant between-group differences were observed for fat and fiber intake at either 3 or 6 months.

### 3.5. Nutrient Adequacy at 3 and 6 Months

**Figure 2:**
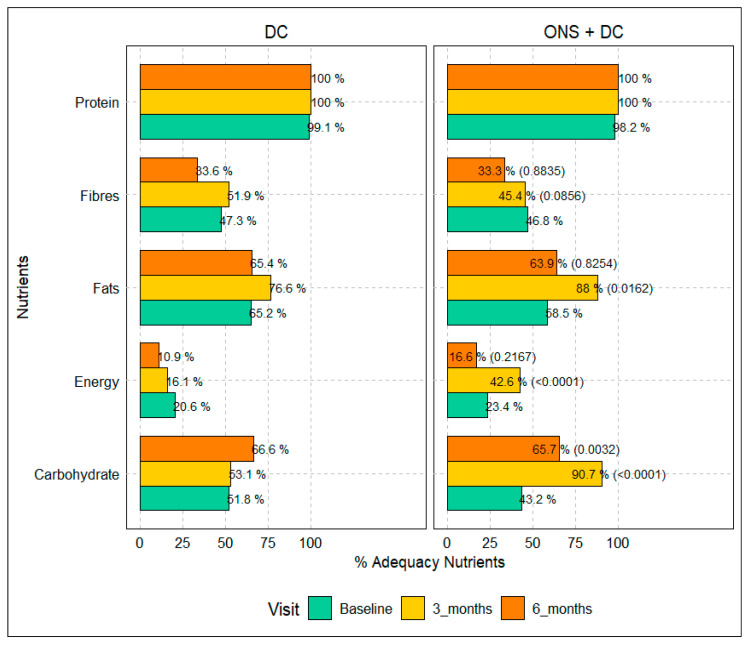
Percentage of adequate subjects by nutrient, visit, and treatment. Note: “% of participants in each group achieving adequacy (*p*-value for between-group comparison at time point)”.

Figure 2 shows nutrient adequacy rates across three visits in the DC and ONS + DC groups. At baseline (screening visit), adequacy was low for energy, carbohydrate, and fat intake, with only 20.5 vs. 23.4%, 43.2 vs. 51.8%, and 58.6 vs. 65.2% of subjects having adequate intakes, respectively, in both groups.

Across all visits, both intervention arms demonstrated improvements in nutrient adequacy; however, the group receiving ONS + DC consistently showed greater improvements compared to the DC alone group. Protein adequacy reached 100% by Visit 3 (3 months) in both groups and was maintained through Visit 4 (6 months). Notably, subjects in the ONS + DC group exhibited significantly higher adequacy percentages for energy and carbohydrates at Visits 3 (3 months) and 4 (6 months) (energy adequacy: 42.5% vs. 17.1% and 16.6% vs. 11.0%; carbohydrate adequacy: 90.7% vs. 53.1% and 66.6% vs. 48.1%, respectively; all *p* < 0.05). Fat adequacy also improved more substantially in the ONS + DC group during these visits. Fiber adequacy showed modest improvement in both groups, without a consistent trend favoring either intervention.

### 3.6. Adverse Events (AEs)

In the ONS + DC group, 67 subjects reported 142 adverse events, while the DC group had 62 subjects with 118 events. The most frequently observed AEs across both groups included fever, cold, cough, headache, body pain, and other mild symptoms. All events were classified as mild in severity, with no reports of life-threatening or fatal outcomes. None of the adverse events reported had a causal relationship with the IP. No participants in either group needed to discontinue or adjust the investigational product due to adverse events, and all events resolved or led to recovery.

### 3.7. IP Compliance

Compliance with the nutritional supplement was high, with a mean of 97.66% and a median of 100%.

## 4. Discussion

This randomized controlled trial demonstrated that ONS, when combined with DC, significantly improved linear growth among children at risk of undernutrition compared to DC alone. The intervention led to notable catch-up growth, particularly in height-for-age, weight status, weight-for-age, and MUACP indices, underscoring the potential of ONS in addressing childhood stunting.

### 4.1. Height Indices

Height-for-age percentile improved significantly in the ONS + DC group, indicating a rapid catch-up in linear growth. Linear catch-up growth in height-for-age is critical in addressing stunting and its associated long-term developmental and health challenges. This improvement is consistent with prior studies showing that ONS contributes to linear growth by providing essential macro- and micronutrients critical during growth in early childhood. Huynh et al. (2015) reported that long-term ONS use led to sustained improvements in height-for-age percentiles, supporting the role of targeted supplementation in promoting linear growth [28]. Similarly, Ow et al. (2024) found that 120 days of ONS combined with dietary counselling significantly improved linear growth in at-risk children [29].

Unlike earlier Indian ONS studies [27,38], the present trial showed a statistically significant improvement in height, which may be attributed to differences in study design and intervention. This study used a fixed, higher-dose regimen (two servings daily) and an enhanced formulation containing arginine, vitamin K2, and casein phosphopeptide, nutrients known to support skeletal growth through improved growth hormone secretion and mineral absorption [39,40,41,42].

### 4.2. Weight and Anthropometric Indices

A significant improvement in weight-for-age percentile observed in the ONS + DC group at both 3 and 6 months (*p* < 0.0001) highlights the positive impact of ONS + DC in promoting weight catch-up growth among children at risk of undernutrition. Previous studies support our findings. Alarcon et al. (2003), Zhang et al. (2021), and Khanna et al. (2021) all demonstrated that ONS with DC improves weight and growth outcomes in undernourished children, aligning with the results of our study [20,25,27].

However, while weight-for-height percentile improved in the ONS + DC group, the differences compared to the DC group were not statistically significant. This suggests that while ONS contributes to weight gain, the increase appears to occur proportionately with height gain, resulting in the maintenance of BMI rather than an excessive rise, suggesting that the supplementation supported balanced growth compared to the control group. Interestingly, though, there were no differences in BMIAP.

The significant improvement in Mid-Upper Arm Circumference (MUAC)-for-age percentile in the ONS + DC group (*p* < 0.0001) suggests that MUAC-for-age percentile may be influenced more by linear growth than associated with BMI-for-age percentile. This is in line with previous studies that suggest MUAC is sensitive to height or the presence of stunting [43,44], and the large change in MUACP in the ONS + DC group observed in this study may reflect height gain.

### 4.3. Sick Days and Sick Episodes

Although the differences were not statistically significant, the observed reduction in sick episodes in the ONS + DC group compared to the DC group suggests a potential benefit of oral nutritional supplementation in supporting immune function. These findings may be influenced by external factors such as environmental exposures, baseline health status, adherence to the intervention, and socioeconomic conditions, including hygiene and living environments, which could have moderated the effect of the intervention on morbidity outcomes [38,45].

### 4.4. Macronutrient and Energy Intake Improvements

The observed improvements in protein and carbohydrate intake in the ONS + DC group reflect the macronutrient composition of the supplement itself, which was designed to provide balanced amounts of macronutrients aligned with the Acceptable Macronutrient Distribution Range (AMDR). Two servings of ONS per day provided approximately 12.8 g of protein (12.5% of total energy), 58.9 g of carbohydrates (57%), and 13.65 g of fat (30%). At baseline, participants’ diets were characterized by relatively low protein intake (~10% of total energy) and a higher proportion of energy from fat (~36%), with carbohydrates accounting for ~54%. The higher protein-to-energy ratio of the ONS compared to baseline intake explains the significant increase in protein intake and the improved protein-energy ratio observed in the intervention group. Furthermore, as the fat contribution of the supplement (30%) was lower than the participants’ baseline fat intake, this likely explains the absence of a significant increase in fat consumption. ONS improved macronutrient balance by increasing protein and carbohydrate intake while lowering fat intake. These changes support growth and align with findings that high-protein supplements enhance outcomes in undernourished children [18,46].

Interestingly, energy intake declined from 3 to 6 months in both groups, with a greater decline in the ONS + DC group, resulting in a diminished and non-significant difference between groups at 6 months. One reason could be the longer interval between the visits at 3 and 6 months and the lack of reinforcement or reminders of DC advice provided between visits. Adequate energy intake is essential for supporting growth, metabolic activities, and physical activity, particularly in children with higher energy demands or those at risk of undernutrition [47,48,49]

### 4.5. Nutrient Adequacy

This study demonstrated that the combined intervention of ONS + DC was more effective than DC alone in improving the nutrient adequacy of key macronutrients, particularly energy, carbohydrates, and fats, over four consecutive visits. The significant gains in energy and carbohydrate adequacy observed in the ONS + DC group reflect the nutrient-dense composition of the supplement, which likely improved overall caloric intake. Sheng et al. also found that supplements with dietary counselling improved nutrient adequacy and growth in picky eaters, including weight-for-age and BMI z-scores [50].

### 4.6. Public Health Relevance

This study highlights the value of combining ONS with DC to combat childhood undernutrition, especially in low-resource settings. ONS + DC was more effective than DC alone and may help reduce stunting and wasting if integrated into national nutrition programs [4]. A key strength of this study is its randomized controlled design with a 6-month follow-up, allowing robust evaluation of both linear and ponderal growth. The use of standardized anthropometric measures, high compliance with ONS, and dietary intake assessments strengthen the reliability of findings.

### 4.7. Study Limitations

This study has a few limitations; it was conducted in a single urban location with children aged 3–6.9 years, which may limit the generalizability of the results to other regions or age groups. Additionally, the open-label design, where both participants and researchers were aware of treatment allocation, may have influenced reporting of subjective outcomes such as dietary intake and health status. The six-month intervention period, while sufficient to observe significant changes, was relatively short, and longer-term follow-up would be beneficial to assess the sustainability of the results. Despite these limitations, the study provides valuable insights into the effectiveness of oral nutritional supplementation combined with dietary counseling in improving the growth and nutritional status of children at nutritional risk.

Another limitation is that compliance with ONS intake was based on parental recall and returned packaging, as the supplementation was administered at home without direct observation. This method is susceptible to recall bias and may have led to an overestimation of actual intake. Moreover, the possibility of other family members, especially siblings, consuming the product cannot be ruled out. In certain cases, it was also observed that regular dietary intake was reduced in comparison to baseline, suggesting the possibility of ONS displacing the regular diet. The apparent discrepancy between reported compliance and estimated nutrient intake changes suggests that not all children may have consistently consumed the full recommended servings, despite recorded high compliance. Emphasizing the fact that ONS should not be replacing the regular dietary intake but instead should be consumed in addition to a regular diet as a mid-morning or mid-afternoon snack could possibly have reduced the gap seen in dietary intake values.

DC alone did not significantly improve dietary intakes in either group, aligning with prior studies showing limited impact without supplementation. A systematic review noted DC improves dietary quality but is less effective alone [15], and the clinical trial found ONS plus DC outperformed DC alone [29]. A limitation was the school-based setting, where time constraints limited DC depth and caregiver involvement, potentially reducing effectiveness. Other factors, such as limited access to diverse, nutrient-rich foods, economic constraints, and inconsistent food availability, can hinder families’ to implement recommended dietary changes. Future studies should include intensive DC with caregiver engagement and strategies to improve ONS adherence.

Future research should explore the long-term impact of ONS on growth, cognitive development, and school performance. Multicentric studies across diverse populations are needed to improve generalizability. Additionally, evaluating biochemical markers, body composition, and cost-effectiveness would provide deeper insights into the mechanisms and scalability of ONS interventions. Further studies should also examine optimal duration, age of initiation, and formulation enhancements tailored to specific nutritional deficiencies.

## 5. Conclusions

This study demonstrates the efficacy of oral nutritional supplementation combined with dietary counseling in improving dietary intake and increasing improvements in anthropometric measures with time, particularly height-for-age percentile, weight-for-age percentile, and MUAC-for-age percentile, without excess or disproportionate weight gain, and shows a reduction in stunting among children at risk of undernutrition.

## Figures and Tables

**Table 1:** Summary of demographic and baseline characteristics.

Parameters	ONS + DC (N = 111)	DC (N = 112)	Total (N = 223)
Gender			
Male	59 (53.2%)	53 (47.3%)	112 (50.2%)
Female	52 (46.8%)	59 (52.7%)	111 (49.8%)
Age (Years)			
Mean (SD)	4.9 (0.9)	4.9 (0.9)	4.9 (0.9)
Height (cm)			
Mean (SD)	101.0 (5.8)	101.7 (5.6)	101.4 (5.7)
Body weight (Kg)			
Mean (SD)	14.0 (1.4)	14.006 (1.3)	14.0 (1.3)
BMI (kg/m^2^)			
Mean (SD)	13.6 (0.5)	13.5 (0.6)	13.5 (0.5)
Height-for-age percentile			
Mean (SD)	7.3 (6.5)	9.0 (7.0)	8.1 (6.7)
Weight-for-height percentile			
Mean (SD)	13.0 (6.4)	12.4 (6.7)	12.8 (6.5)

Note: N = total number of subjects in treatment group. n = total number of non-missing subjects in specified category. SD = standard deviation.

**Table 2:** Summary of change in mean height and weight at 3 and 6 months from baseline.

Parameters	Visits	Statistics	ONS + DC (N = 111)	DC (N = 112)	Difference [95% CI]	*p*-Value
Average Height (cm)	Baseline	n	111	112	-	<0.001 *
		Mean (SD)	101.0 (5.8)	101.7 (5.7)	-	
	Visit 3 (3 M)	n	108	111	-	
		Mean (SD)	104.8 (5.7)	103.3 (5.6)	-	
	CFB Visit 3 (3 M)		3.9 (1.5)	1.6 (0.1)	2.3 [1.9, 2.6]	
	Visit 4 (6 M)	n	108	110	-	<0.001 *
		Mean (SD)	106.8 (5.7)	104.1 (5.9)	-	
	CFB Visit 4 (6 M)		5.8 (1.5)	2.4 (1.0)	3.4 [3.1, 3.8]	
Average Body Weight (kg)	Baseline	n	111	112	-	<0.001 *
		Mean (SD)	14.0 (1.3)	14.0 (1.3)	-	
	Visit 3 (3M)	n	108	111	-	
		Mean (SD)	15.5 (1.7)	14.9 (1.6)	-	
	CFB Visit 3 (6 M)		1.6 (0.8)	0.9 (0.8)	0.7 [0.5, 0.9]	
	Visit 4 (6 M)	n	108	110	-	<0.001 *
		Mean (SD)	16.0 (1.6)	15.0 (1.6)	-	
	CFB Visit 4 (6 M)		2.1 (0.9)	0.9 (0.8)	1.1 [0.9, 1.3]	

Abbreviations: N = total number of subjects in treatment group; n = total number of non-missing subjects in specified category; SD = standard deviation; CFB = change from baseline; (CFB = Post baseline–baseline); LS Mean = least-squares mean; ONS = oral nutritional supplement; DC = dietary counseling. Note: *p*-value has been derived from two samples independent *t*-test. * *p*-values with asterisks are *p* < 0.05.

**Table 3:** Effect of ONS intervention on anthropometric parameters from baseline to 3 months and 6 months.

Parameters	Visits	ONS + DC (N = 111)		DC (N = 112)		LS Mean Difference [95% CI]	*p*-Value
		n	Mean (SD)	n	Mean (SD)		
Height-for-Age Percentile (HAP)	Baseline	111	7.3 (6.5)	112	9.0 (6.9)	-	-
	At 3 months	108	17.2 (15.7)	111	10.4 (9.0)	-	<0.0001 *
	CFB at 3 months	-	10.0 (10.8)	-	1.3 (4.8)	9.50 [7.4, 11.5]	
	At 6 months	108	19.4 (16.0)	110	8.4 (8.1)	-	<0.0001 *
	CFB at 6 months	108	12.1 (11.3)	110	−0.4 (4.8)	13.3 [11.1, 15.4]	
Height-for-Age Z-Score (HAZ)	Baseline	111	−1.7 (0.5)	112	−1.5 (0.5)	-	-
	At 3 months	108	−1.1 (0.6)	111	−1.4 (0.6)	-	<0.0001 *
	CFB at 3 months	-	0.5 (0.3)	-	0.04 (0.2)	0.5 [0.4, 0.6]	
	At 6 months	108	−1.0 (0.6)	110	−1.6 (0.6)	-	<0.0001 *
	CFB at 6 months	-	0.6 (0.3)	-	−0.09 (0.2)	0.7 [0.6, 0.8]	
Weight-for-Height Percentile (WHP)	Baseline	59	13.9 (6.4)	61	12.4 (6.7)	-	–
	At 3 months	50	26.1 (19.5)	54	22.1 (18.8)	-	0.5017
	CFB at 3 months	-	12.6 (16.8)	-	9.9 (15.6)	2.1 [−4.0, 8.2]	
	At 6 months	44	26.3 (19.0)	43	18.7 (16.9)	-	0.1103
	CFB at 6 months	-	12.6 (17.2)	-	6.6 (13.9)	5.33 [−1.2, 11.9]	
Weight-for-Height Z-Score (WHZ)	Baseline	59	−1.2 (0.3)	61	−1.2 (0.4)	-	–
	At 3 months	50	−0.7 (0.6)	54	−0.9 (0.7)	-	0.9406
	CFB at 3 months	-	0.3 (0.5)	-	0.3 (0.5)	0.1 [−0.2, 0.3]	
	At 6 months	44	−0.7 (0.6)	43	−1.0 (0.7)	-	0.2706
	CFB at 6 months	-	0.3 (0.5)	-	0.1 (0.5)	0.21 [−0.1, 0.5]	
BMI for Age Percentile	Baseline	111	11.0 (7.16)	112	10.3 (7.1)	-	–
	At 3 months	108	21.9 (18.0)	111	19.8 (17.5)	-	0.9012
	CFB at 3 months	-	10.8 (15.2)	-	9.4 (14.4)	1.3 [−3.7, 6.4]	
	At 6 months	108	20.6 (18.3)	110	18.0 (16.4)	-	0.7605
	CFB at 6 months	-	9.4 (15.8)	-	7.5 (13.6)	1.9 [−3.1, 7.0]	
BMI for Age Z-Score	Baseline	111	−1.3 (0.4)	112	−1.41 (0.5)	-	–
	At 3 months	108	−0.9 (0.6)	111	−1.0 (0.7)	-	0.8520
	CFB at 3 months	-	−0.9 (0.6)	-	−1.0 (0.7)	0.1 [−0.1, 0.3]	
	At 6 months	108	−1.00 (0.7)	110	−1.1(0.7)	-	0.5525
	CFB at 6 months	-	−1.00 (0.7)	-	−1.1 (0.7)	0.1 [−0.1, 0.2]	
Weight for Age Percentile (WAP)	Baseline	112	3.9 (4.0)	112	4.3 (4.1)	-	–
	At 3 months	108	13.0 (13.4)	111	8.6 (9.5)	-	<0.0001 *
	CFB at 3 months	-	9.0 (10.5)	-	4.2 (7.1)	5.3 [2.5, 8.2]	
	At 6 months	108	13.7(14.9)	110	6.7 (8.1)	-	<0.0001 *
	CFB at 6 months	-	9.7 (11.3)	-	2.3 (6.3)	7.9 [5.0, 10.7]	
Weight-for-Age Z-Score (WAZ)	Baseline	112	−1.9 (0.5)	112	−1.92 (0.5)	-	–
	At 3 months	108	−1.3 (0.6)	111	−1.66 (0.6)	-	<0.0001 *
	CFB at 3 months	-	0.6 (0.4)	-	0.2 (0.4)	0.3 [0.2, 0.5]	
	At 6 months	108	−1.3 (0.6)	110	−1.8 (0.6)	-	<0.0001 *
	CFB at 6 months	-	0.6 (0.4)	-	0.1 (0.4)	0.5 [0.4, 0.7]	
MUAC-for-Age Percentile (MUACP)	Baseline	59	11.2 (8.3)	61	11.4 (8.4)	-	-
	At 3 months	50	20.6 (17.6)	54	14.4 (11.0)	-	0.0165 *
	CFB at 3 months	-	9.8 (13.6)	-	3.0 (7.6)	6.5 [0.8, 12.1]	
	At 6 months	44	21.7 (16.1)	43	10.4 (9.4)	-	<0.0001 *
	CFB at 6 months	-	11.1 (13.0)	-	−1.05 (8.4)	11.1 [5.28, 16.9]	
MUAC-for-Age Z-Score (MUACZ)	Baseline	59	−1.3 (0.5)	61	−1.3(0.5)	-	-
	At 3 months	50	−0.9 (0.6)	54	−1.2 (0.5)	-	0.0113 *
	CFB at 3 months	-	0.4 (0.5)	-	0.1 (0.3)	0.2 [0.1, 0.5]	
	At 6 months	44	−0.8 (0.5)	43	−1.4 (0.6)	-	<0.0001 *
	CFB at 6 months	-	0.5 (0.5)	-	−0.1 (0.5)	0.6 [0.3, 0.8]	

Abbreviations: N = total number of subjects in treatment group; n = total number of non-missing subjects in specified category. SD = standard deviation; CFB = change from baseline; (CFB = Post baseline–baseline); LS Mean = least-squares mean; ONS = oral nutritional supplement; DC = dietary counseling; MUACP = mid-upper arm circumference percentile; MUACZ= mid-upper arm circumference Z score. Note: The mean difference was estimated using an analysis of covariance adjusted for randomization stratification factors: study center, gender, treatment*gender, age (numeric), and baseline value. * *p*-values with asterisks are *p* < 0.05. The *p*-value was derived using an ANOVA test.

**Table 4:** Number of sick days and sick episodes at 3 and 6 months.

Parameter Visits	Statistics	ONS + DC (N = 111)	DC (N = 112)
Number of Sick Days			
Visit 3 (3 Months)	n	34	24
	Mean (SD)	3.8 (1.7)	3.7 (1.5)
	Average Sick Rate	1.17	0.79
	Rate Ratio [95% CI]	1.04 [0.7, 1.3]	
	*p*-value	0.7845	
Visit 4 (6 Months)	n	22	31
	Mean (SD)	3.6 (1.1)	3.2 (1.3)
	Average Sick Rate	0.71	0.8
	Rate Ratio [95% CI]	1.12 [0.83, 1.52]	
	*p*-value	0.4470	
Number of Sick Episodes			
Visit 3 (Month 3)	E (%) n	60 (30.6) 34	37 (21.4) 24
	Rate Ratio [95% CI]	1.16 [0.7, 1.7]	
	*p*-value	0.4918	
Visit 4 (Month 6)	E (%) n	45 (19.8) 22	49 (27.7) 31
	Rate Ratio [95% CI]	1.24 [0.8, 1.8]	
	*p*-value	0.3008	

Note: N = total number of subjects in treatment group; n = number of subjects who experienced an event in specified treatment group; E = number of episodes. The *p*-value was derived using an ANOVA test. The rate ratio is based on the negative binomial model adjusted for randomization stratification factors study center, gender, treatment*gender, and age (numeric).

**Table 5:** Change in calorie intake at 3 and 6 months compared to baseline.

Parameter	Visit	ONS + DC (N = 111) (n = 108), Mean, (SD)	DC (N = 112) (n = 110), Mean, (SD)	LS Mean Difference [95% CI]	*p*-Value
Carbohydrates (g/day)	Baseline	149.0, (36.7)	150.3, (35.4)	-	-
	3 Months	180.3, (26.4)	151.9, (29.5)	-	-
	CFB at 3 Months	31.1 (39.9)	1.6 (44.2)	28.5 [19.3, 37.7]	<0.0001 *
	6 Months	163.5, (25.6)	151.2, (25.9)	-	-
	CFB at 6 Months	14.3 (40.0)	0.74 (38.9)	12.4 [3.2, 21.6]	0.0031 *
Fats (g/day)	Baseline	44.5, (18.9)	46.1, (17.4)	-	-
	3 Months	49.0, (10.4)	46.4, (13.7)	-	-
	CFB at 3 Months	4.8 (20.4)	0.4 (21.1)	2.7 [−1.0, 6.4]	0.2466
	6 Months	42.8, (10.1)	43.4, (10.0)	-	-
	CFB at 6 Months	−1.41 (22.0)	−2.6 (21.4)	−0.4 [−4.2, 3.2]	0.9883
Protein (g/day)	Baseline	27.7, (8.8)	29.3, (9.6)	-	-
	3 Months	38.2, (9.0)	31.9, (9.1)	-	-
	CFB at 3 Months	10.58 (11.7)	2.75 (11.3)	6.4 [3.6, 9.3]	<0.0001 *
	6 Months	33.0, (7.7)	29.2, (6.9)	-	-
	CFB at 6 Months	5.42 (12.5)	0.004 (11.2)	4.10 [1.23, 6.96]	0.0014 *
Energy (Kcal/day)	Baseline	1106.1, (294.4)	1127.7, (282.1)	-	-
	3 Months	1309.3, (166.9)	1155.64, (199.6)	-	-
	CFB at 3 Months	206.5 (307.0)	29.3 (337.9)	155.9 [96.3, 215.4]	<0.0001 *
	6 Months	1174.7, (173.1)	1123.1, (166.8)	-	-
	CFB at 6 Months	71.87 (330.1)	−4.70 (324.9)	54.2 [−5.4, 113.9]	0.0897
Protein/ Energy Ratio (g/Kcal)	Baseline	0.03, (0.01)	0.03, (0.01)	-	-
	3 Months	0.03, (0.01)	0.03, (0.01)	-	-
	CFB at 3 Months	0.004 (0.007)	0.0014 (0.008)	0.0017 [−0.0003, 0.0037]	0.1294
	6 Months	0.03, (0.01)	0.03, (0.01)	-	-
	CFB at 6 Months	0.003 (0.009)	−0.0003 (0.008)	0.0022 [0.0003, 0.0042]	0.0204
Fibres (g/day)	Baseline	15.0, (5.6)	15.7, (5.3)	-	-
	3 Months	15.5, (4.5)	16.3, (4.7)	-	-
	CFB at 3 Months	0.34 (6.0)	0.6 (6.3)	−0.6 [−2.2, 0.8]	0.6619
	6 Months	14.4, (3.5)	14.6, (4.9)	-	-
	CFB at 6 Months	−0.8 (5.8)	−1.06 (7.0)	−0.1 [−1.6, 1.3]	0.9942

Abbreviations: N = total number of subjects in treatment group; n = total number of non-missing subjects in specified category. SD = standard deviation; CFB = change from baseline; (CFB = post baseline – baseline); LS Mean = least-squares mean. Note: The mean difference was estimated using a repeated measures analysis of covariance adjusted for randomization stratification factors study center, gender, treatment*gender, visit, treatment*visit, age (numeric), and baseline value. * *p*-values with asterisks are *p* < 0.05. The *p*-value was derived using an ANOVA test.

## Data Availability

The data presented in this study are available on request from the corresponding author.

## Supplementary Materials

The following supporting information can be downloaded at: https://www.mdpi.com/article/10.3390/children12091152/s1, Table S1: Nutritional composition of ONS for 45.5 g of powder; Table S2: Sensitivity analyses excluding outliers in height change from baseline—Change in anthropometric growth parameters at 3 and 6 months of outcomes derived from height measurements.

## Abbreviations

The following abbreviations are used in this manuscript:

ONSs	Oral Nutritional Supplements
DC	Dietary Counseling
BMI	Body Mass Index
HAP	Height-For-Age Percentiles
WHP	Weight-For-Height Percentiles
WAP	Weight-For-Age Percentiles
MUAC	Mid-Upper Arm Circumference
WHO	World Health Organization
DXA	Dual-Energy X-ray Absorptiometry
