# Supplementary material for: Impact of Oral Nutritional Supplementation and Dietary Counseling on Outcomes of Linear Catch-Up Growth in Indian Children Aged 3–6.9 Years: Findings from a 6-Month Randomized Controlled Trial
Source: Children (Basel). 2025 Aug 29;12(9):1152. doi: 10.3390/children12091152 (PMC12468868; doi:10.3390/children12091152)
Supplement: Supplementary file 1 [file children-12-01152-s001.zip › Supplementary Table S1.pdf]

Supplementary Table S1: Nutritional composition of ONS for 45.5 g of powder

**Nutrient**	**Unit**	**Per 100 g of Powder**	**Per Serving* (45.5 g of powder)**
Energy	kcal	452	205.7
Protein	g	14.1	6.4
Fat	g	15	6.8
Carbohydrate	g	64.74	29.5
FOS	g	1.58	0.7
Taurine	mg	28	12.7
Carnitine	mg	6.7	3.0
Inositol	mg	32	14.6
Arginine	mg	1099	500.0
Vitamin A	mcg RE	439	199.7
Vitamin D_2	mcg	11	5.0
Vitamin E	mg a-TE	7.7	3.5
Vitamin K_1	mcg	26.3	12.0
Vitamin K_2	mcg	8.8	4.0
Vitamin C	mg	35.2	16.0
Folic acid	mcg	110	50.1
Vitamin B_1	mg	0.9	0.4
Vitamin B_2	mg	1	0.5
Vitamin B_6	mg	1	0.5
Vitamin B_{12}	mcg	1.09	0.5
Niacin	mg NE	7	3.2
Pantothenic acid	mcg	3100	1410.5
Biotin	mcg	16	7.3
Choline	mg	118	53.7
Sodium	mg	175	79.6
Potassium	mg	512	233.0
Chloride	mg	420	191.1
Calcium	mg	605	275.3
Phosphorus	mg	408	185.6

Magnesium	mg	50	22.8
Iron	mg	12.09	5.5
Zinc	mg	4.94	2.2
Manganese	mg	0.98	0.4
Copper	mcg	400	182.0
Iodine	mcg	99	45.0
Selenium	mcg	32.9	15.0
Chromium	mcg	12	5.5
Molybdenum	mcg	19.7	9.0
Standard reconstitution is 203 g/L.<br>A single serving is prepared by mixing 45.5 g of powder with 190 mL of water, for a final volume of 225 mL.			
