# Supplementary material for: Impact of Oral Nutritional Supplementation and Dietary Counseling on Outcomes of Linear Catch-Up Growth in Indian Children Aged 3–6.9 Years: Findings from a 6-Month Randomized Controlled Trial
Source: Children (Basel). 2025 Aug 29;12(9):1152. doi: 10.3390/children12091152 (PMC12468868; doi:10.3390/children12091152)
Supplement: Supplementary file 1 [file children-12-01152-s001.zip › Supplementary Table S2.pdf]

Supplementary Table S2: Sensitivity analyses excluding outliers in height change from baseline - Change in anthropometric growth parameters at 3 and 6 months of outcomes derived from height measurements.

**Parameters**<br>**Visits**	**Statistics**	**ONS + DC (N=102)**	**DC-only (N=112)**
Average Height (cm)			
Baseline	n	102	112
	Mean (SD)	101.26 (5.792)	101.70 (5.631)
CFB of Average Height (cm)			
Visit 3 (3 Months)	n	99	111
	Mean (SD)	3.67 (1.269)	1.63 (0.974)
	Diff [95% CI]	-2.04 [-2.35, -1.72]	
	p-value*	<0.001	
Visit 4 (6 Months)	n	99	110
	Mean (SD)	5.62 (1.288)	2.41 (1.094)
	Diff [95% CI]	-3.21 [-3.53, -2.88]	
	p-value*	<0.001	
Height-for-Age Z-score (+/-)			
Baseline	n	102	112
	Mean (SD)	-1.659 (0.5515)	-1.526 (0.5628)
CFB of Height-for-Age Z-score (+/-)			
Visit 3 (3 Months)	n	99	111
	Mean (SD)	0.479 (0.2742)	0.042 (0.2096)
	LS Mean difference [95% CI]	0.44 [0.36, 0.53]	
	p-value	<0.0001	
Visit 4 (6 Months)	n	99	110
	Mean (SD)	0.589 (0.2749)	-0.088 (0.2370)
	LS Mean difference [95% CI]	0.69 [0.60, 0.77]	

	p-value	<0.0001	
Height-for-Age Percentile			
Baseline	n	102	112
	Mean (SD)	7.225 (6.4114)	8.996 (6.9059)
CFB of Height-for-Age Percentile			
Visit 3 (3 Months)	n	99	111
	Mean (SD)	8.553 (9.1346)	1.388 (4.8069)
	LS Mean difference [95% CI]	8.07 [6.28, 9.85]	
	p-value	<0.0001	
Visit 4 (6 Months)	n	99	110
	Mean (SD)	10.782 (9.8941)	-0.461 (4.8776)
	LS Mean difference [95% CI]	11.96 [9.99, 13.94]	
	p-value	<0.0001	
Weight-for-Height Z-score (+/-)			
Baseline	n	52	61
	Mean (SD)	-1.200 (0.3767)	-1.246 (0.4240)
CFB of Weight-for-Height Z-score (+/-)			
Visit 3 (3 Months)	n	43	54
	Mean (SD)	0.337 (0.5511)	0.332 (0.5660)
	LS Mean difference [95% CI]	-0.01 [-0.31, 0.29]	
	p-value	0.9998	
Visit 4 (6 Months)	n	37	43
	Mean (SD)	0.358 (0.5892)	0.190 (0.5111)
	LS Mean difference [95% CI]	0.17 [-0.14, 0.48]	

	p-value	0.4786	
Weight-for-Height Percentile			
Baseline	n	52	61
	Mean (SD)	13.139 (6.4322)	12.432 (6.7174)
CFB of Weight-for-Height Percentile			
Visit 3 (3 Months)	n	43	54
	Mean (SD)	11.403 (16.4176)	9.911 (15.6166)
	LS Mean difference [95% CI]	0.34 [-5.64, 6.32]	
	p-value	0.9100	
Visit 4 (6 Months)	n	37	43
	Mean (SD)	12.385 (17.9609)	6.696 (13.9419)
	LS Mean difference [95% CI]	4.28 [-2.40, 10.95]	
	p-value	0.2056	
BMI-for-Age Z-score (+/-)			
Baseline	n	102	112
	Mean (SD)	-1.362 (0.4571)	-1.417 (0.5319)
CFB of BMI-for-Age Z-score (+/-)			
Visit 3 (3 Months)	n	99	111
	Mean (SD)	0.385 (0.5382)	0.340 (0.5418)
	LS Mean difference [95% CI]	0.05 [-0.15, 0.25]	
	p-value	0.9084	
Visit 4 (6 Months)	n	99	110
	Mean (SD)	0.305 (0.5444)	0.241 (0.5493)
	LS Mean difference [95% CI]	0.07 [-0.13, 0.27]	
	p-value	0.7881	

BMI-for-Age Percentile			
Baseline	n	102	112
	Mean (SD)	10.760 (7.1078)	10.353 (7.1693)
CFB of BMI-for-Age Percentile			
Visit 3 (3 Months)	n	99	111
	Mean (SD)	10.476 (14.9559)	9.410 (14.4183)
	LS Mean difference [95% CI]	1.02 [-4.07, 6.11]	
	p-value	0.9547	
Visit 4 (6 Months)	n	99	110
	Mean (SD)	8.796 (15.1142)	7.577 (13.6727)
	LS Mean difference [95% CI]	1.23 [-3.87, 6.33]	
	p-value	0.9242	

Outliers were identified using the interquartile range method, based on changes in height (centimetres) from baseline at each visit. Nine outliers with large height gain values in the ONS+DC group were identified, and excluded, in the sensitivity analyses.

*p-value has been derived from two samples independent t-test.

Abbreviations:
N = Total number of subjects in treatment group; n = Total number of non-missing subjects in specified category; SD = Standard Deviation; CFB = Change from baseline (Post baseline – Baseline); LS Mean = Least squares mean; CI = Confidence interval; ONS = Oral nutritional supplement; DC = Dietary counselling.

Note: The LS mean difference was estimated using a repeated measure analysis of covariance adjusted for randomization stratification factors study centre, gender, treatment*gender, visit, treatment*visit, age(numeric) and baseline value.
